# Rat Hypocretin/Orexin Neurons Are Maintained in a Depolarized State by TRPC Channels

**DOI:** 10.1371/journal.pone.0015673

**Published:** 2010-12-16

**Authors:** Vesna Cvetkovic-Lopes, Emmanuel Eggermann, Aaron Uschakov, Jeremy Grivel, Laurence Bayer, Barbara E. Jones, Mauro Serafin, Michel Mühlethaler

**Affiliations:** 1 Département de Neurosciences Fondamentales, Centre Médical Universitaire, Genève, Switzerland; 2 Department of Neurology and Neurosurgery, McGill University, Montreal Neurological Institute, Montreal, Quebec, Canada; Pennsylvania State University, United States of America

## Abstract

In a previous study we proposed that the depolarized state of the wake-promoting hypocretin/orexin (hcrt/orx) neurons was independent of synaptic inputs as it persisted in tetrodotoxin and low calcium/high magnesium solutions. Here we show first that these cells are hyperpolarized when external sodium is lowered, suggesting that non-selective cation channels (NSCCs) could be involved. As canonical transient receptor channels (TRPCs) are known to form NSCCs, we looked for TRPCs subunits using single-cell RT-PCR and found that TRPC6 mRNA was detectable in a small minority, TRPC1, TRPC3 and TRPC7 in a majority and TRPC4 and 5 in the vast majority (∼90%) of hcrt/orx neurons. Using intracellular applications of TRPC antibodies against subunits known to form NSCCs, we then found that only TRPC5 antibodies elicited an outward current, together with hyperpolarization and inhibition of the cells. These effects were blocked by co-application of a TRPC5 antigen peptide. Voltage-clamp ramps in the presence or absence of TRPC5 antibodies indicated the presence of a current with a reversal potential close to −15 mV. Application of the non-selective TRPC channel blocker, flufenamic acid, had a similar effect, which could be occluded in cells pre-loaded with TRPC5 antibodies. Finally, using the same TRPC5 antibodies we found that most hcrt/orx cells show immunostaining for the TRPC5 subunit. These results suggest that hcrt/orx neurons are endowed with a constitutively active non-selective cation current which depends on TRPC channels containing the TRPC5 subunit and which is responsible for the depolarized and active state of these cells.

## Introduction

The hypothalamic hypocretin/orexin (hcrt/orx) neurons are critical for maintaining a waking state (for reviews, see [1]–[4]). Giving rise to widespread projections throughout the brain [5], they play a central role in promoting waking through the excitatory actions of their peptide upon multiple arousal systems [6]–[10]. During the natural sleep-wake cycle, they discharge during waking and in association with behavioral arousal [11], [12].

In a previous study [13], we showed *in vitro* that hcrt/orx neurons were spontaneously active and maintained in a depolarized state. We then demonstrated that this state did not depend upon voltage-dependent sodium channels or synaptic activity as it was not modified in the presence of tetrodotoxin (TTX) and/or a low Ca^2+^/high Mg^2+^ solution. Evidence that cesium also did not affect the depolarized state indicated that an I_h_ current was not implicated either. Although the mechanism underlying the depolarized state could not be established at that time, we speculated that it could be due to the presence of a non-selective cation current.

Several channels have been shown to carry non-selective cation currents (see for review [14]) and could thus be candidates to conduct the current that maintains hcrt/orx cells in their depolarized state. Among them, are the seven members of the “canonical” transient receptor potential (TRPC) subfamily [15], which belongs to a wider superfamily of cation-permeable TRP channels [16], [17]. The role of TRPC channels in maintaining a depolarized state in neurons was recently demonstrated in GABAergic neurons of the substantia nigra pars reticulata (SNr) where TRPC3 channels were implicated [18].

In the present study, we thus hypothesized that the depolarized state of hcrt/orx neurons might also depend upon the presence of constitutively active TRPC channels. Our results, while indicating that this is indeed the case, differ from those in the SNr [18]. In hcrt/orx neurons, TRPC channels containing the TRPC5 subunit are involved and these channels have properties which are quite different from those in the SNr.

## Results

### The depolarized state of hcrt/orx neurons is due to a non-selective cation current

**Figure 1:**
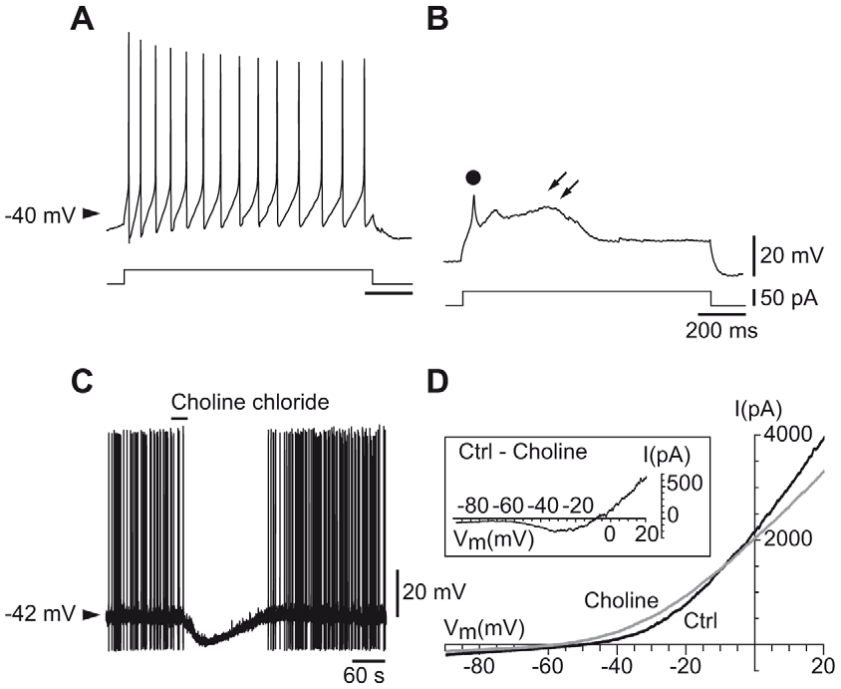
Hyperpolarization of hcrt/orx neurons by substitution of sodium with choline chloride. (A–B) Tonic firing in presence of a depolarizing step (A) and a low-threshold spike (dot) followed by plateau potential (double arrow) when the depolarizing step is given under DC hyperpolarization (B) is characteristic of hcrt/orx neurons. (C) Substitution of sodium chloride with choline chloride in the bath produces a hyperpolarization and cessation of firing. (D) Voltage-clamp ramps in control (Ctrl) conditions and after choline substitution. The subtraction of ramps shown in the inset suggests the presence of a voltage-dependent cationic current.

Hcrt/orx neurons were identified by the usual criteria [13]. In short, a depolarizing step applied at rest yielded tonic firing (Fig. 1A), whereas in condition of membrane hyperpolarization, it yielded a depolarizing response characterized by the successive presence of a low-threshold spike (LTS, dot in Fig. 1B) and a plateau potential (arrows in Fig. 1B). Such neurons were previously identified by immunohistochemistry as expressing hcrt/orx [13].

In a preceding *in vitro* study [13], all hcrt/orx neurons were found to be in a depolarized state which was affected by neither low calcium, nor TTX, nor cesium (to block the I_h_ current). We can add here that while nickel (100 µM, n = 3) had no effect on the resting potential, the calcium channel blocker cadmium (0.5 to 1.0 mM; in 0.1 mM calcium) slightly hyperpolarized the cells (mean ΔV ± SEM  = −3.7±1.0 mV, n = 5; not shown). We then hypothesized that the depolarized state could be due to the presence of a non-selective cation current having sodium as a major charge carrier [18]. We therefore tested whether lowering sodium in the bath from 150 to 20 mM, by substituting sodium chloride with choline chloride, would affect the membrane potential. As shown in Fig. 1C, neurons immediately hyperpolarized (mean ± SEM  = −11.55±0.53 mV, n = 4) in this condition. Subtracting voltage-clamp ramps obtained in control (ACSF) with ramps obtained after sodium substitution (Fig. 1D) then allowed us to characterize the current underlying the depolarized state (inset of Fig. 1D). As illustrated below (with respect to the effect of a TRPC antibody or flufenamic acid, FFA), this current exhibits a strong voltage-dependence with a region of negative slope and shows a reversal potential around −5 mV (mean ± SEM  = −6.1±0.95 mV, n = 3) compatible with it being a non-selective cation current.

Finally it should be noted that although removing calcium from the ACSF did not affect the membrane potential and blocking voltage-dependent calcium channels only had a minimal effect, a basic level of intracellular calcium was needed to maintain the depolarized state. Indeed, intracellular application of BAPTA (20 mM instead of 0.1 mM) strongly hyperpolarized the cells (mean ΔV ± SEM  = −15.3±1.4 mV, n = 7). Altogether these results suggest the presence of NSCCs in hrct/orx neurons.

### TRPC subunits expressed in hcrt/orx neurons

**Figure 2:**
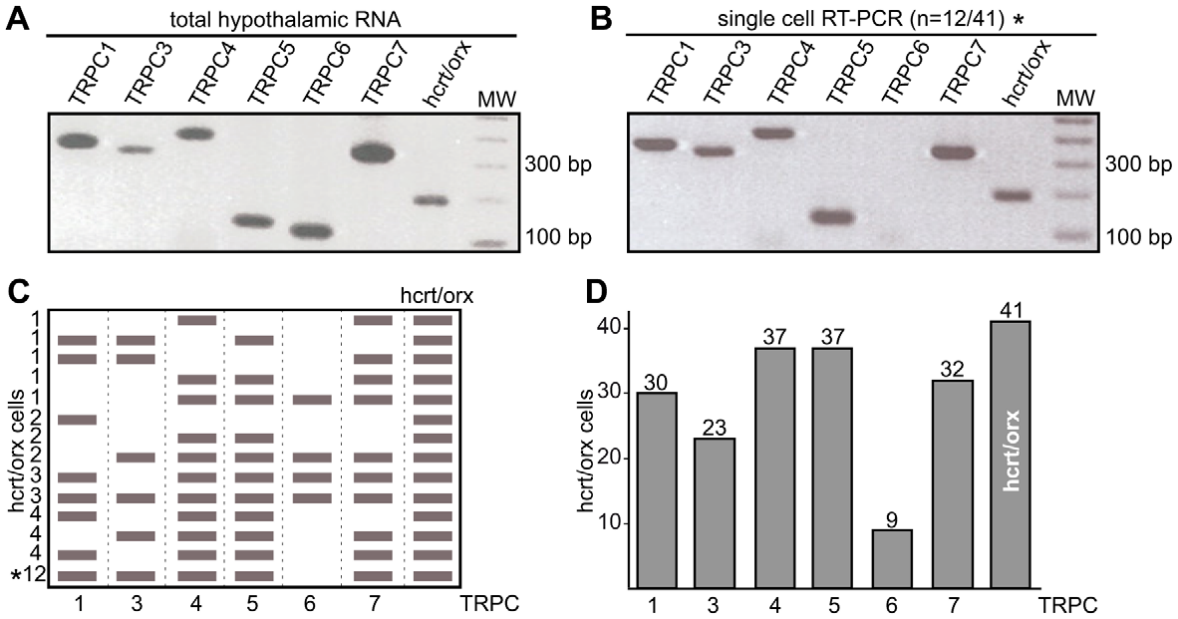
Single-cell RT-PCR of TRPCs in hcrt/orx neurons. (A) RT-PCR experiment on 500 pg total RNAs isolated from the hypothalamus. Agarose gel electrophoresis showing that mRNAs encoding the TRPC1, 3, 4, 5, 6, and 7 are all detected and generate PCR fragments with sizes of respectively 363, 333, 402, 129, 114 and 337 bp. The hcrt/orx PCR fragment size is 189 bp. MW: molecular weight (100 bp DNA ladder). (B) Single-cell RT-PCR experiment. Agarose gel electrophoresis illustrating the more frequently observed TRPC expression pattern, found in 12 out of 41 hcrt/orx cells. It corresponds to TRPC1, 3, 4, 5 and 7. MW: molecular weight (100 bp DNA ladder). (C) Different TRPC expression patterns identified in the 41 hcrt/orx cells investigated. The asterisk indicates the more frequently observed pattern also illustrated in panel B. (D) Histogram showing the occurrence of the different TRPC subunits in the 41 investigated cells.

Since NSCCs can be composed of TRPC subunits (see Discussion) and given the recent evidence of substantia nigra neurons being maintained in an intrinsic depolarized state by TRPC channels [18], we investigated the possible involvement of TRPCs in the depolarized state of hcrt/orx neurons. For that purpose, we studied first whether these neurons express TRPC subunits using single-cell multiplex RT-PCR. The RT-PCR conditions were determined using 100 to 500 pg of total RNAs purified from hypothalamic slices. In these conditions, six TRPC subunits (i.e. TRPC1 and TRPC3-7; TRPC2 not being reported for reasons exposed in the Methods) along with hcrt/orx transcripts were successfully detected, each generating a PCR fragment of the size predicted by its mRNA sequence (Fig. 2A). The identity of the fragments was confirmed by sequencing.

Single-cell RT-PCR was then used to investigate the expression of TRPC subunits in 41 hcrt/orx cells, which were identified by their electrophysiological properties. Hcrt/orx cDNA was successfully amplified in all neurons and, depending on the TRPC subunit present, a number of different expression patterns were identified. The main pattern was characterized by the presence of TRPC1, 3, 4, 5 and 7 (illustrated on Fig. 2B) and was found in 12 (out of 41) hcrt/orx neurons (* in Fig. 2C), whereas other patterns (Fig. 2C) were found less frequently, some of them being present in only one cell. Taken together, as illustrated in Fig. 2D, only TRPC4 and 5 were present in almost every cell (37/41) and were thus considered as the prime candidates for a role in the tonic depolarization characterizing all hcrt/orx neurons.

### Hyperpolarization of hcrt/orx neurons by a TRPC5 antibody

Because we found a majority of hcrt/orx neurons expressing the TRPC4 and 5 subunits, we investigated their function by using intracellular applications of specific antibodies (6 ng/µl) against TRPC4 and 5 subunits. With the same approach, we also examined the potential involvement of the TRPC3 subunit (found in 23 of 41 cells), since it was shown to be involved in the tonic depolarization of SNr GABAergic neurons [18], and the TRPC1 subunit (found in 30 of 41 cells), since it can co-assemble with a TRPC5 subunit to form a functional hetero-multimere channel [19].

**Figure 3:**
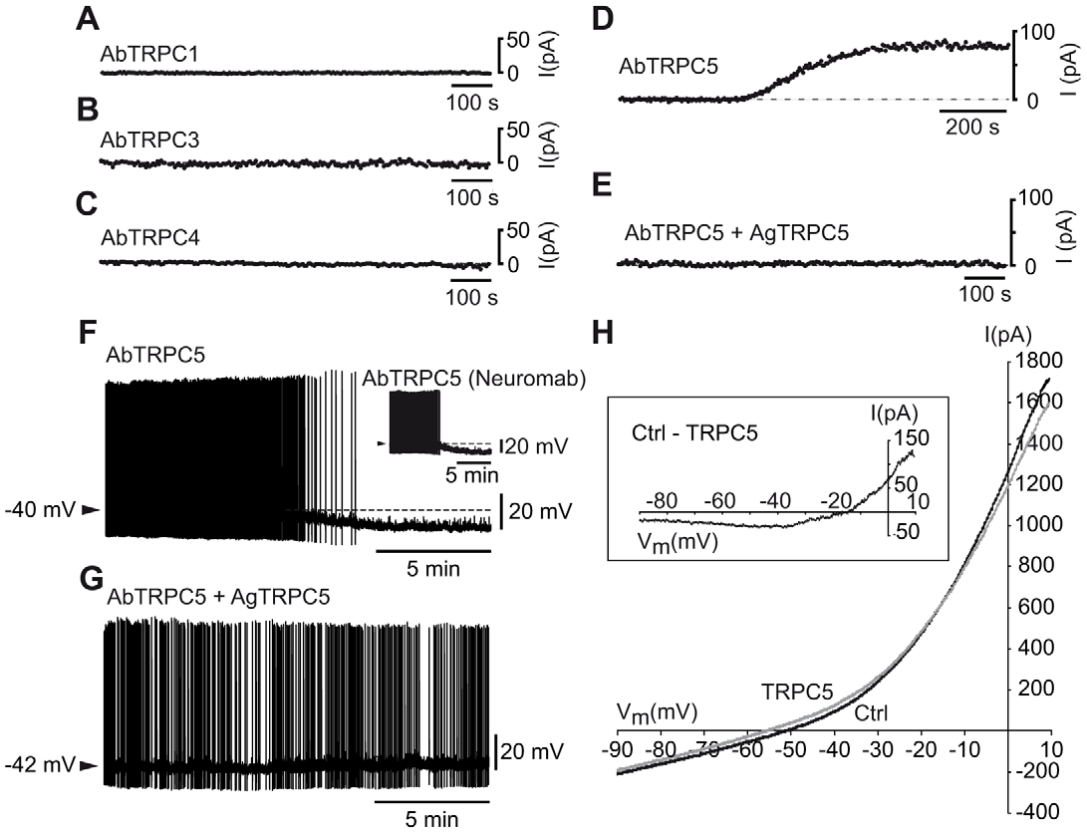
The depolarized state of hcrt/orx neurons is blocked by a TRPC5 antibody. (A–C) In voltage-clamp configuration, intracellular application of either a TRPC1 (A) or a TRPC3 (B) or a TRPC4 (C) antibody (AbTRPC1, AbTRPC3 and AbTRPC4 respectively, all from Alomone Labs) has no effect on the current needed to clamp the cell at −50 mV. (D–E) Intracellular application of a TRPC5 antibody (Alomone Labs), in contrast, induces an outward current (D) that is blocked by co-application of a TRPC5 antigen (E). (F–G) In current-clamp configuration, intracellular application of a TRPC5 antibody alone (from either Alomone Labs (F) or NeuroMab (inset in F) induces an important membrane hyperpolarization and cessation of firing. In contrast, intracellular co-application of the TRPC5 antibody (Alomone Labs) together with its appropriate antigen (AgTRPC5) has no effect (G, to compare with F). (H) Voltage-clamp ramps in absence (Ctrl) and presence of a TRPC5 antibody (AbTRPC5, Alomone Labs). In the inset, subtraction of voltage-clamp ramps (Ctrl - AbTRPC5) suggests the presence of a voltage-dependent TRPC5 current.

Intracellular application of TRPC1,3 or 4 antibodies on voltage-clamped cells, yielded no change in the current needed to maintain them at −50 mV (TRPC1, Fig. 3A, mean ΔI ± SEM  = −0.76±2.78 pA, n = 5; TRPC3, Fig. 3B, mean ΔI  = 1.78±1.67 pA, n = 8; TRPC4, Fig. 3C, mean ΔI  = −2.60±1.91 pA, n = 6).

In contrast, application of the TRPC5 antibody (Fig. 3D) produced a persistent outward current (mean ΔI ± SEM  = 77.0±15.2 pA, n = 9). This response was specific as it could be blocked (Fig. 3E) by co-application of a TRPC5 antigen (used at 12 or 18 ng/µl) with the TRPC5 antibody (mean ΔI  = −1.38±1.38 pA, n = 4). In current-clamp, the TRPC5 antibody hyperpolarized (Fig. 3F) and completely inhibited the cells (mean ΔV ± SEM  = −11.16±0.73 mV, n = 10) together with an elimination of the plateau potential (n = 3/3, not shown). The hyperpolarizing action was antagonized (Fig. 3G; mean ΔV  = −0.33±0.20 mV, n = 4) by co-application of the antibody with the corresponding antigen. The hyperpolarizing action of the TRPC5 antibody (from Alomone Labs) was checked with an antibody from a different source (NeuroMab, used in [20] also applied intracellularly at 6 ng/µl which yielded similar results (inset of Fig. 3F, mean ΔV ± SEM  = −12.08±1.56 mV, n = 4; Student's t test Alomone versus NeuroMab p = 0.62).

Finally it should be stressed that in 3/3 non-hcrt/orx neurons (according to their electrophysiological properties) held at −50 mV, application of the TRPC5 antibody yielded no change of membrane current (not shown).

### Hcrt/orx neurons are depolarized by active TRPC channels

The above data suggest that a non-selective cation current, depending on the presence of channels containing the TRPC5 subunit, could maintain the neurons in their depolarized state. To further substantiate this view, we studied first hcrt/orx cells with voltage-clamp ramps in presence of TTX. As illustrated in Fig. 3H (inset), subtraction of a control ramp obtained immediately after whole-cell is established with one obtained a few minutes later, as the TRPC5 antibody diffuses into the cell, indicates the presence of a current with a strong voltage-dependence and a region of negative slope. Its reversal potential close to −15 mV (mean ± SEM  = −17.43±0.52 mV, n = 3) is compatible with it being a non-selective cation current. At rest, which in hcrt/orx cells is close to −45 mV [13], this current must thus contribute to their depolarized state and spontaneous activity. The fact that this current is active at rest might explain why lanthanum (100 µM), which is known as a positive modulator of TRPC5 channels [19], had no effect here (n = 3/3).

**Figure 4:**
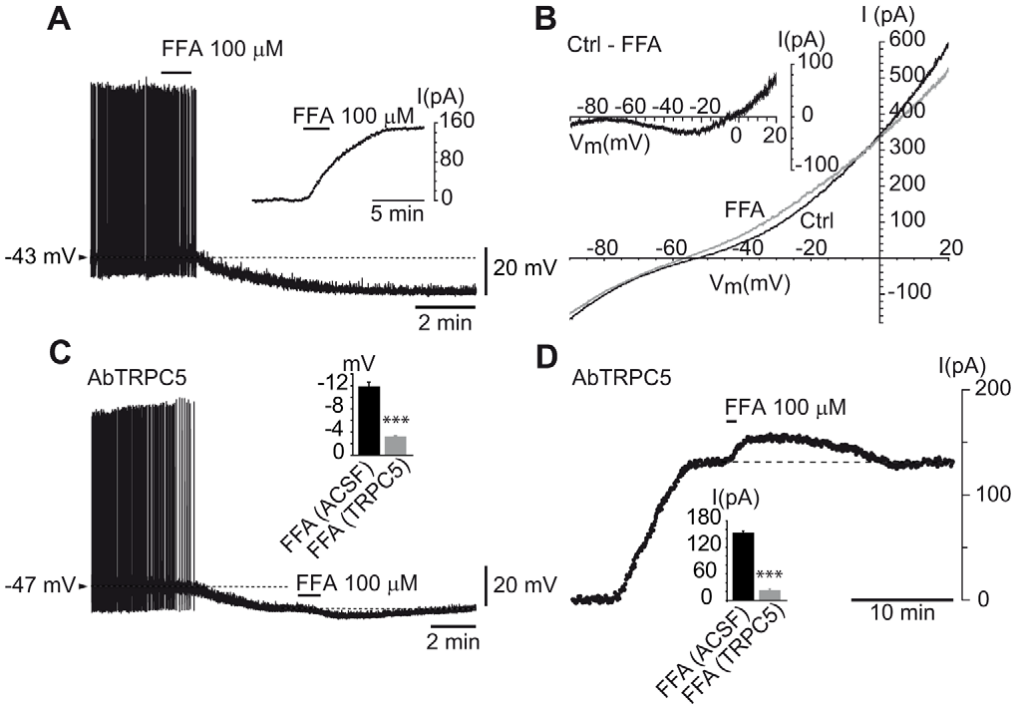
The TRPC5 antibody occludes the effect of FFA in hcrt/orx neurons. (A) Transient (2 min) bath-application of 100 µM flufenamic acid (FFA), a non specific blocker of cationic currents, produces a strong membrane hyperpolarization and cessation of firing; inset: in voltage-clamp this hyperpolarization corresponds to an outward current. (B) Voltage-clamp ramps in absence (Ctrl) and presence (FFA) of flufenamic acid. The subtraction of voltage-clamp ramps shown in the inset suggests the presence of a voltage-dependent cationic current. (C–D) In hcrt/orx neurons loaded with a TRPC5 antibody, bath-application of FFA had only a small additional effect either in current-clamp (C) or in voltage-clamp (D).

To further substantiate the suggestion that TRPCs are involved in maintaining the depolarized state of hcrt/orx neurons, we tested the effect of the non-selective TRPC blocker, flufenamic acid (FFA). As illustrated in Fig. 4A, application of FFA (100 µM), induced a hyperpolarization (mean ΔV ± SEM  = −11.88±0.72 mV, n = 4) or an outward current (inset of Fig. 4A; mean ΔI ± SEM  = 152.5±2.5 pA, n = 3). Using voltage-clamp ramps, it can be seen (inset of Fig. 4B) that the subtracted current has a shape similar to the one observed with sodium chloride substitution or the TRPC5 antibody. The mean reversal potential was also in the same range (mean E_inv_ (FFA) ± SEM  = −10.43±2.31 mV, n = 3). To determine whether the TRPC5 antibody and FFA share the same target, we applied FFA in cells that were pre-loaded with the antibody. As shown in Fig. 4C, following the usual hyperpolarization by the antibody, application of FFA had only a small hyperpolarizing effect left (mean ΔV ± SEM  = −3.29±0.08 mV, n = 4) that is a 70.3% reduction of the FFA effect (Student's t test comparing the effect of FFA in absence versus presence of TRPC5 antibody in the pipette; p = 0.001). Similar results were obtained in voltage-clamp, in presence of TTX, where again FFA applied in cells pre-loaded with the antibody, showed only a minimal effect (mean ΔI ± SEM  = 23.5±2.85 mV, n = 3) that is a 84.6% reduction of the FFA effect (Student's t test comparing the effect of FFA in absence versus presence of TRPC5 antibody in the pipette p<0.001).

The TRPC-dependent current observed here could depend on the presence of transmitters, released tonically by neurons in the slice in a calcium-independent manner [21]. As transmitters are thought to activate TRPC channels [16], [22] via an increase in phospholipase C (PLC), as illustrated notably in the action of glutamate [23] and acetylcholine [19], [24], we tested this hypothesis using long-lasting (at least 20 min) perfusion with U-73122, a PLC inhibitor, but found that cells never hyperpolarized with this treatment (n = 3, not shown).

### Immunohistochemical demonstration of TRPC5 subunits in hcrt/orx neurons

**Figure 5:**
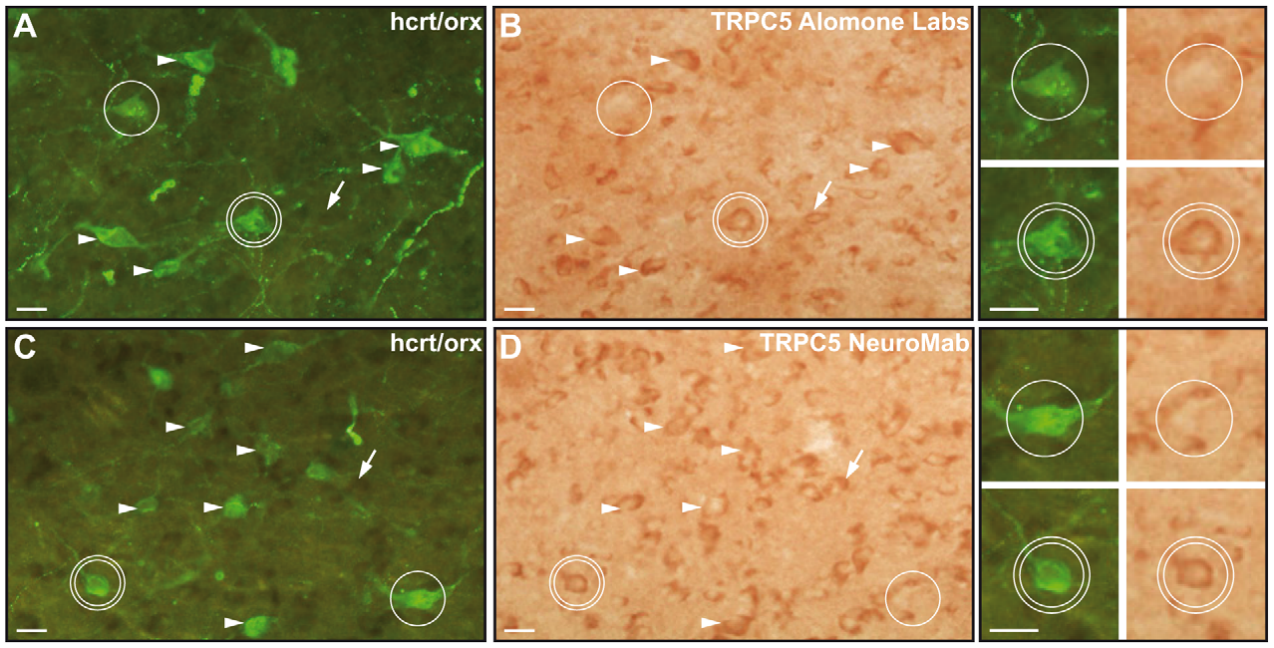
Dual immunohistochemical staining for TRPC5 and hcrt/orx. (A–B) Photomicrographs from a dual-immunostained section combining an hcrt/orx antibody from Santa Cruz (A) and a TRPC5 antibody from Alomone Labs (B). Examples of double labelled hcrt/orx-TRPC5 cells are indicated by white arrowheads and one such cell by a double circle (enlarged in the corresponding small panels to the right). Some hcrt/orx cells do not express TRPC5, as shown by an example indicated with a single circle. It is noteworthy that many hcrt/orx-negative cells also expressed TRPC5, as shown by an example indicated with a downward arrow (in panels A and B). (C–D) Same as in A and B with an hcrt/orx antibody from Phoenix Pharmaceuticals (C) and a TRPC5 antibody from NeuroMab (D). (Scale bars: 20 µm).

The TRPC5 subunit expression in hcrt/orx cells was verified by dual immunohistochemical staining combining immunoperoxidase staining for TRPC5 and immunofluorescence staining for hcrt/orx. Immunostaining for hcrt/orx with the two antibodies employed revealed cytoplasmic staining of multiple neurons through the perifornical hypothalamus (Fig. 5A,C), as previously described [25]. With the two antibodies employed for TRPC5 (from Alomone Labs and NeuroMab), peroxidase staining of the cytoplasm of multiple cells was evident through the perifornical hypothalamus (Fig. 5B,D). The TRPC5 labelling was observed in many hcrt/orx-positive cells (arrowheads in Fig. 5A–D and double circles magnified in images on the right) and also hcrt/orx-negative cells (examples indicated by downward going arrows in Fig. 5A–D) of varying sizes. As illustrated in Fig. 5A–D (single circle) some hcrt/orx cells did not show TRPC5 staining with either antibody. We found 78.7% (with Alomone labs) and 70.6% (with NeuroMab TRPC5 antibodies) of hcrt/orx cells to be immunopositive for TRPC5.

## Discussion

From our previous *in vitro* work in which we discovered that hcrt/orx neurons are in a depolarized and active state independent of synaptic inputs [13], we postulated that this state could be due to the presence of NSCCs. Here, we provide evidence that the membrane depolarization and spontaneous activity are maintained by a non-selective cation current depending on the presence of TRPC channels containing TRPC5 subunits.

That the depolarized state of hcrt/orx neurons depends on the presence of a non-selective cation current is based on several arguments. First, we previously demonstrated [13] that the depolarized state could not depend on voltage-dependent sodium channels or synaptic inputs since it persisted in presence of TTX and/or a low Ca^2+^/high Mg^2+^ solution. Further evidence that the depolarized state does not depend critically on voltage-dependent calcium channels was reported here since nickel had no effect and cadmium only a minimal one. The depolarized state depended however on a basal level of intracellular calcium since loading cells with BAPTA led to a strong membrane hyperpolarization as we report here. Second, we previously found [13] that the depolarized state could not depend on the I_h_ current since cesium would not lead to membrane hyperpolarization either. Third, in the present study, we found in contrast that substituting sodium chloride with choline chloride immediately led to a hyperpolarization (or outward current in voltage-clamp) of the cells. Fourth, the reversal potential of the tonic current removed by the sodium substitution was around −5 mV, which fits with a non-selective cation current.

That the non-selective current was dependent on TRPC channels relies on several arguments. First, we show here that the hcrt/orx neurons can be hyperpolarized (or show an outward current) in presence of flufenamic acid, a blocker of TRPC channels (for reviews [14], [16]). The reversal potential of the current removed by FFA was similar to the one removed by sodium substitution. Although FFA is not specific for TRPCs, as it interferes with a number of other channels [14], it provides preliminary evidence of a role for TRPCs. Second, the single-cell RT-PCR investigation demonstrated that mRNAs for many TRPC subunits are present in hcrt/orx cells and that for at least two of them, TRPC4 and 5, they are present in the vast majority (∼90%) of the cells. Third, by using specific antibodies against the intracellular part of different TRPC subunits, as recently used in mammalian neurons of the amygdala [23] and substantia nigra [18], we could show that TRPC5 subunits are implicated in the depolarized state of hcrt/orx cells. Indeed, application of a TRPC5 antibody induced a hyperpolarization (or outward current) similar to that seen with FFA or sodium chloride substitution, including a similar reversal potential. The effect of the TRPC5 antibody was specific as it was blocked when the TRPC5 antigen peptide was co-applied. Fourth, the effect of FFA could be occluded by pre-loading of the cells with the antibody, indicating that both experiments involve the same target. Finally, immunohistochemical staining performed with the same TRPC5 antibodies which were used in the physiological experiments showed that the TRPC5 subunits are present in 70 to 80% of hcrt/orx cells.

The TRPC channels are part of a 7-member subfamily of non-selective cation channels [15], which is itself part of the TRP superfamily that comprises six families of cation-permeable channels [16], [17]. Depending on the TRPC subtype and the tissue involved, TRPC channels were shown to be activated by a variety of stimuli (including neurotransmitters) or to be constitutively active. More specifically and relevant to our study, constitutively active TRPC3 channels have recently been shown to be responsible for the depolarized and active state of GABAergic neurons in the SNr [18], following the earlier demonstration of TRPC3 channels playing a similar role in rabbit ear arterial myocytes [26]. Our results in hcrt/orx neurons also suggest the presence of constitutively active TRPC channels, given the hyperpolarizing action of sodium chloride substitution, FFA, and a TRPC antibody, coupled to the lack of effect of blocking synaptic transmission or impeding phospholipase C dependent pathways [22]. Our results however differ from those in SNr neurons as TRPC5 subunits are implicated here and, in addition, the current described in the present study has a strong voltage-dependence, not very different from the one seen in HEK cells co-transfected with TRPC1 and TRPC5 cDNAs [19]. In our study, application of a TRPC1 antibody was however ineffective, leaving the possibility open that a heteromultimer of TRPC5 and TRPC1 subunits is at play here. Others [23] had encountered similar difficulties, not being able to reduce metabotropic responses to glutamate with a TRPC1 antibody alone (while a TRPC5 antibody worked) and yet having a voltage-dependence of the current typical for the TRPC1/TRPC5 heteromultimer. Of final notice, the voltage-dependence of the current in hcrt/orx neurons and its sensitivity to intracellular calcium could explain the presence of the plateau potential and its boosting by the low-threshold calcium spike [13].

Functionally, these results support the hypothesis that the natural state of hcrt/orx neurons is depolarized and spontaneously active. This intrinsic property is likely important for the role of hcrt/orx neurons in promoting and maintaining wakefulness since their excitatory influence upon the multiple arousal systems [5]–[10] could be maintained without exogenous input. Their decreased activity during sleep [11], [12] should thus mainly depend upon the GABAergic inhibitory input they receive from sleep-promoting neurons located in the preoptic area and basal forebrain [13], [27]–[30].

In conclusion, our results indicate that hcrt/orx neurons are endowed with a non-selective cation current carried by TRPC channels, which are constitutively open at the resting potential of these cells, and thereby contribute to their depolarized and active state.

## Methods

### Slice preparation and electrophysiological recordings

Hypothalamic coronal brain slices (250 µm thick) containing the hcrt/orx neurons were obtained from young Sprague-Dawley rats (18–21 days) reared at the animal facility of the Geneva Medical Center or obtained from Charles-River Laboratories (France) and treated according to the rules of the Swiss Federal Veterinary Office. The study was reviewed and approved by the “office vétérinaire cantonal” (***approval ID: 31.1.1007/3248/0***). Before use, slices were incubated at room temperature in artificial cerebrospinal fluid (ACSF) containing in (mM): NaCl 130, KCl 5, KH_2_PO_4_ 1.25, MgSO_4_ 1.3, NaHCO_3_ 20, glucose 10 and CaCl_2_ 2.4 (bubbled with 95% O_2_ and 5% CO_2_). Individual slices were then transferred to a thermoregulated (32°C) recording chamber mounted on an upright microscope (Axioskop, Zeiss, Oberkochen, Germany) equipped with an infrared camera (TILL-Photonics, Gräfelfing, Germany). Slices were maintained immersed and continuously superfused at 4–5 ml/min with ACSF.

Whole-cell recordings were obtained with patch electrodes (3–5 MΩ) that were pulled on a DMZ universal puller (Zeitz-Instrumente, Munich, Germany) from borosilicate capillaries (GC150F-10, Harvard instruments, UK). The pipettes were filled with a solution containing (in mM): KMeSO_4_ 126, KCl 4, MgCl_2_ 5, BAPTA 0.1 (or EGTA 0.05), HEPES 10, phosphocreatine 8, ATP 3, GTP 0.1, pH 7.3, 285–300 mOsm. High BAPTA experiments were done with electrodes having the same composition with the exception of BAPTA raised to 20 mM and KMeSO_4_ lowered to 86 mM (the results of BAPTA experiments were already reported with respect to the effect on the plateau potentials [13] but not with respect to the influence on resting potentials, which are thus reported here). Whole-cell recordings were performed in the current clamp or voltage clamp mode using an Axopatch 200B amplifier (Axon Instruments, Union City, CA). The membrane potential values were not compensated for junction potential (estimated at −9.6 mV). Membrane potentials were around −45 mV (on a sample of 30 cells, mean ± SEM  = −45.94±0.73 mV, n = 30), as previously described [13]. It could be measured easily as firing is irregular in these cells. Membrane resistance was not systematically checked but on a random sample of recordings it was around 500 MΩ (mean ± SEM  = 525.23±48.96 MΩ, n = 10).

Voltage-clamp experiments were all done in presence of tetrodotoxin (TTX, 10^−6^ M, Latoxan, France) with series resistance (usual range from 10 to 20 MΩ) monitored continuously by short-lasting hyperpolarizing steps to −50 mV every 5 seconds (not visible on the figure traces as voltage/current plots are sampled outside the steps by the analysis/display software). Experiments were discarded if series resistance varied more than 20%. In some voltage-clamp experiments, IV relations were obtained with voltage ramps (duration 500 ms to 6 s; delivered every 5 to 60 s) from +10, +20 or +50 mV to −90 mV (decreasing ramps used to inactivate calcium currents).

For experiments using intracellular application of TRPC antibodies (see below), the pipettes were first tip-filled with a pipette solution devoid of TRPC antibody and then back-filled with 3.5 µl of a pipette solution containing the tested TRPC at a final concentration of 6 ng/µl. When used, the TRPC5 control antigen (Alomone Labs) was added (at 12 or 18 ng/µl) to the TRPC5 antibody in the pipette.

### Chemicals

Most chemicals were obtained from Sigma-Aldrich (Buchs, Switzerland), with the exception of the PLC inhibitor, U-73122 from Tocris (Lucernachem, Lucerne, Switzerland). To avoid precipitation, lanthanum (100 µM) was used in an ACSF containing in mM: NaCl 150, KCl 6.25, Hepes 11, glucose 10, MgCl_2_, 1.3, sucrose 18 and CaCl_2_ 2.4. For experiments with choline chloride, 130 mM of NaCl were substituted with 130 mM of choline chloride in the ACSF.

When testing TRPCs antibodies, those for TRPC1, 3 and 4 were obtained from Alomone Labs (Jerusalem, Israel), whereas the one for TRPC5 was obtained from either Alomone Labs or NeuroMab (UC Davis, California, USA). References and validation (based on physiology, western blotting, immunohistochemistry, antisense and siRNA approaches) for the Alomone antibodies used in the present study (i.e. for TRPC1, 3, 4 and 5) can be found in the following papers: for TRPC1 [31]–[36]; for TRPC3 [18], [26], [35], [37], [38]; for TRPC4 [34]–[36], [39]; for TRPC5 [31], [35], [39]–[41]. For validation of the TRPC5 NeuroMab antibody, see [20].

### Single-cell RT-PCR

The multiplex PCR conditions were first optimized using total RNA purified (RNA Now, Ozyme, Saint-Quentin-en-Yvelines, France) from hypothalamic slices containing hcrt/orx neurons (250 µm thick, from a 19 days-old rat), so that all TRPC subunits and hcrt/orx transcripts could be detected from 100 to 500 pg of total RNA, and were then applied to the single cell studies. The identities of the amplified fragments were confirmed by sequencing.

Following electrophysiological identification of the neuron (Fig. 1A,B see Eggermann et al., 2003), the cell content was aspirated under visual control into the patch pipette containing 8 µl of intra-pipette solution. The pipette content was immediately expelled into a tube containing 20 U of RNase inhibitor (Stratagene, Basel, Switzerland) for blocking RNase activity and 20 U of DNase I (Qiagen, Courtaboeuf, France) for digestion of genomic DNA. Samples were incubated at 37°C for 30 min, followed by incubation at 75°C for 5 min to inactivate the DNase. The different cDNAs were then synthesized overnight at 37°C in a total reaction volume of about 15 µl containing random hexamer primers (3.33 µM; Roche Applied Science, Rotkreuz, Switzerland), the four deoxyribonucleotide triphosphates (333 µM; Invitrogen, Lucerne, Switzerland), dithiothreitol (6.6 mM; Sigma, Buchs, Switzerland), RNase inhibitor (20 U, Stratagene), and reverse transcriptase (Superscript II; 200 U; Invitrogen). The cDNAs for the different TRPC subunits and for hcrt/orx were first simultaneously amplified in a multiplex PCR reaction using degenerate (Dg) primers [42], [43] for TRPC subunits and the following primers (from 5′ to 3′) for hcrt/orx (189 bp): sense TGC CGT CTC TAC GAA CTG TTG CAC G, antisense AGG GAT ATG GCT CTA GCT CTG CGC C.

Multiplex PCR was performed in a final reaction volume of 50 µl containing the totality of the RT reaction, Herculase Hotstart DNA polymerase (2.5 U; Stratagene), deoxyribonucleotide triphosphates (0.2 mM each; Invitrogen), 1 µM of each Dg primers and 50 nM of each hcrt/orx primers with the following cycling protocol: after 5 min at 94°C, 35 cycles (94°C for 48 s, 50°C for 1 min, 72°C for 90 s) were run followed by a final elongation period of 10 min at 72°C. In a second round of PCR, each TRPC subunit and hcrt/orx cDNAs were specifically amplified (using 1 µM of each TRPC nested primers and 0.1 µM of each hcrt/orx primers) in individual reactions with 2 µl of the multiplex PCR product under similar conditions for 35 cycles (94°C for 30 s, 52°C for 45 s, 72°C for 2 min). The PCR products (15 µl) were analyzed in ethidium bromide-stained agarose gels (2%).

The primers used for **TRPC1** (363 bp), **TRPC5** (129 bp) and **TRPC6** (114 bp) amplifications were from [44]. For TRPC3 (333 bp), TRPC4 (402 bp) and TRPC7 (337 bp), the following primers were used (from 5′ to 3′): **TRPC3** sense GAC TGT CGA AGA CAT ATT CCA GTT CA, antisense ACA TCA CTG TCA TCC TCG ATT TCT T; **TRPC4** sense [44], antisense GCA AAT TTC CAC TCT ATA TCT GCG TG; **TRPC7** sense GAA CTG TGA AAG ACA TCT TCA AGT T, antisense TCC ACG TCT GCG TCT TCC TCG ATT.

Although TRPC2 expression is restricted to the vomeronasal organ [45] and could have been skipped, we attempted to analyze it in the present study but were unsuccessful. In a preliminary study on total rat hypothalamic RNAs, using two different TRPC2 couples of primers [42], [44] and different RT-PCR protocols, we could not get a clean amplification of a product with the expected length. The bands obtained were sequenced but did not correspond to TRPC2. It is noteworthy that similar problems were encountered by others [23].

Finally, to validate our results, several controls were done: 1) to check for non-specific harvesting of surrounding tissue components, pipettes containing 8 µl of the internal solution were advanced into slices and retrieved without sealing and aspirating before proceeding to RT-PCR protocol. 2) For each PCR amplification, controls for contaminating artifacts were performed using sterile water instead of cDNA. 3) A control consisting of harvesting the content of a hcrt/orx neuron and processing it while omitting the RT was also performed. As expected, all types of controls gave negative results.

### GenBank accession numbers

Hcrt/orx (NM_013179), TRPC1 (NM_053558), TRPC3 (AB022331), TRPC4 (NM_001083115, NM_080396), TRPC5 (NM_080898), TRPC6 (NM_053559), TRPC7 (XM_225159, XM_001067646).

### Double immunohistochemistry for hcrt/orx and TRPC5

Paraformaldehyde-fixed brains from 18-19 days-old rats were serially cut into 30-µm coronal floating sections on a cryostat. A protocol combining immunoperoxidase staining for TRPC5 (adapted from NeuroMab (http://www.neuromab.org/Perox_secs.pdf)) and immunofluorescence staining for hcrt/orx was performed. Sections were first processed for the TRPC5 immunoperoxidase staining using two different antibodies (one from Alomone Labs, rabbit polyclonal, dilution 1∶100 and the other from NeuroMab, mouse monoclonal, clone N67/15, dilution 1∶50). Sections were subsequently immersed for 30 min. in 0.3% H_2_O_2_ in PBS at RT, rinsed in TBS (0.15M NaCl, 50 mM Tris, pH 7.5), 45 min. in 0.5% Triton X-100 in TBS at 4°C, rinsed in TBS, 45 min. in TBS containing 0.1% Triton X-100 and 5% normal serum (from the same species as the secondary antibody, donkey or horse serum) at 4°C and then incubated for 48 h at 4°C in the TRPC5 antibody. Following rinsing in TBS, sections were incubated two hours at 4°C in a secondary biotinylated donkey anti-rabbit IgG (Jackson ImmunoResearch, USA, dilution 1∶200) or biotinylated horse anti-mouse IgG antibody (Vector Laboratories, USA, dilution 1∶200) and processed according to the avidin-biotin complex (ABC) method (Vectastain *Elite* ABC Kit, Vector Laboratories, USA). The sections were developed for peroxidase reactivity with 3,3′-diaminobenzidine (DAB, Sigma-Aldrich, USA) from 5 to 10 minutes until desired stain intensity was obtained. The hcrt/orx staining was secondly revealed using a goat orexin-A antibody (Santa Cruz Biotechnology, Germany, dilution 1∶500) when the rabbit TRPC5 antibody was used or the rabbit orexin-A antibody (Phoenix Pharmaceuticals, Germany, dilution 1∶1000) when the mouse TRPC5 antibody was used. Sections were incubated 30 min in TBS containing 0.1% Triton X-100 and 5% normal donkey serum, then overnight with the orexin antibody at RT, followed by two hours incubation with Alexa-Fluor 488-conjugated donkey anti-goat or anti-rabbit IgG secondary antibody (Invitrogen, Switzerland, dilution 1∶500).

Primary and secondary antibodies were all diluted in a TBS solution containing 0.1% Triton X-100 and 5% normal serum (for TRPC5 step) or 3% normal serum (for hcrt/orx step) (the normal serum was from the same species as the secondary antibody used in each step). Sections were observed with a Zeiss Axioskop 2 microscope equipped with an AxioCam color CCD camera (Zeiss). Images were recorded on a computer through the AxioVision software (Zeiss).

To ensure specificity of the immunohistochemical staining, different control conditions were tested. Sections were incubated without the TRPC5 or the hcrt/orx primary antibody and processed for dual-immunostaining. In these series, no staining was evident for TRPC5 or hcrt/orx in the respective series lacking the primary antibody. Other sections were incubated with the rabbit anti-TRPC5 antibody (Alomone Labs) that had been preincubated with the corresponding control antigen (from Alomone Labs, in a ratio of 1∶3 for 2 hours at room temperature). (The control antigen for the TRPC5 antibody from NeuroMab was not available.) In these sections, the immunostaining for TRPC5 was eliminated by the pre-adsorption of the primary antibody with the antigen.
